# Assessing the clinical utility of genetic risk scores for targeted cancer screening

**DOI:** 10.1186/s12967-020-02699-w

**Published:** 2021-01-22

**Authors:** Carly A. Conran, Zhuqing Shi, William Kyle Resurreccion, Rong Na, Brian T. Helfand, Elena Genova, Siqun Lilly Zheng, Charles B. Brendler, Jianfeng Xu

**Affiliations:** 1 University of Illinois College of Medicine, Chicago, IL USA; 2 Program for Personalized Cancer Care, NorthShore University HealthSystem, Evanston, IL USA; 3 Fudan Institute of Urology, Huashan Hospital, Fudan University, Shanghai, China

**Keywords:** Genetic risk score, Single nucleotide polymorphism, Translational genomics, Cancer screening, Breast cancer, Prostate cancer, Colorectal cancer

## Abstract

**Background:**

Genome-wide association studies have identified thousands of disease-associated single nucleotide polymorphisms (SNPs). A subset of these SNPs may be additively combined to generate genetic risk scores (GRSs) that confer risk for a specific disease. Although the clinical validity of GRSs to predict risk of specific diseases has been well established, there is still a great need to determine their clinical utility by applying GRSs in primary care for cancer risk assessment and targeted intervention.

**Methods:**

This clinical study involved 281 primary care patients without a personal history of breast, prostate or colorectal cancer who were 40–70 years old. DNA was obtained from a pre-existing biobank at NorthShore University HealthSystem. GRSs for colorectal cancer and breast or prostate cancer were calculated and shared with participants through their primary care provider. Additional data was gathered using questionnaires as well as electronic medical record information. A t-test or Chi-square test was applied for comparison of demographic and key clinical variables among different groups.

**Results:**

The median age of the 281 participants was 58 years and the majority were female (66.6%). One hundred one (36.9%) participants received 2 low risk scores, 99 (35.2%) received 1 low risk and 1 average risk score, 37 (13.2%) received 1 low risk and 1 high risk score, 23 (8.2%) received 2 average risk scores, 21 (7.5%) received 1 average risk and 1 high risk score, and no one received 2 high risk scores. Before receiving GRSs, younger patients and women reported significantly more worry about risk of developing cancer. After receiving GRSs, those who received at least one high GRS reported significantly more worry about developing cancer. There were no significant differences found between gender, age, or GRS with regards to participants’ reported optimism about their future health neither before nor after receiving GRS results.

**Conclusions:**

Genetic risk scores that quantify an individual’s risk of developing breast, prostate and colorectal cancers as compared with a race-defined population average risk have potential clinical utility as a tool for risk stratification and to guide cancer screening in a primary care setting.

## Background

As genome-wide association studies (GWASs) have become increasingly important since their conception in 2008, scientists have made substantial progress in the field of genetic epidemiology. Specifically, thousands of disease-associated single nucleotide polymorphisms (SNPs) have been identified. Many of these SNPs may be additively combined to generate polygenic risk scores, or genetic risk scores (GRSs), that confer risk for a specific disease. Although the clinical validity of GRSs to predict risk of specific diseases has been well established in seminal publications, no healthcare system, to our knowledge, has assessed their clinical utility by applying GRSs in a primary care setting for cancer risk assessment and targeted intervention.

At present, the screening recommendations for colorectal, breast, and prostate cancers used in primary care are based on risk assessment models that incorporate age, family history (FH), and varying clinical markers [1–4]. In the era of personalized medicine, however, it has been demonstrated that these risk assessment models can be improved by including genetic information to more accurately estimate an individual’s risk of developing these specific cancers [5–9]. There is a need for this more objective and personalized information, as screening recommendations now rely on divergent guidelines and possibly imprecise, incomplete or unknown FH information. Furthermore, these currently available models are useful only for the minority of individuals who have a positive FH; as most individuals who develop cancer, especially at a young age, do not have a known FH of the disease [9–11].

Through the present clinical trial, GRSs were used clinically as part of a multivariate model that included FH of disease as well as other clinical risk factors, to stratify patients based on their individual genetic risk of developing a disease. Investigators, in consultation with surgical oncology specialists, developed a unique genetic test to identify SNPs as well as implementation models (Additional file 1: Appendix A) to guide more personalized risk stratification and screening for colorectal, breast, and prostate cancers in a primary care setting. However, it was important to assess whether increased worry among patients about the possibility of developing cancer would outweigh the benefits of this gained knowledge.

## Methods

### Design and study population

This clinical study involved primary care patients without a personal history of breast, prostate or colorectal cancers who were 40–70 years old at enrollment. All participants were recruited from an existing IRB-approved genomic study, called the Genomic Health Initiative at NorthShore University HealthSystem, in which DNA samples had already been collected and stored. Participants were re-contacted and provided additional written informed consent for the current study.

The study also enrolled a second group: 59 primary care physicians (PCPs). Here, we will be discussing only the experiences of the 281 participants who were enrolled in the study. Briefly, the Genomic Health Initiative database was queried for individuals who met eligibility criteria. PCPs of the identified individuals were contacted for participation in the physician arm of the study. PCP involvement required watching educational videos about GRSs and confirming comfort with reporting results. Videos included basic genetic information about DNA and risk-associated SNPs, a cancer risk assessment overview involving family history and GRSs, and modified cancer screening guidelines that incorporate GRSs into standard of care for all three cancers. Participants were not contacted until their PCP approved our recruitment of their patients. Ultimately, the number of participants enrolled under individual PCPs ranged from 1 to 21 participants.

Of note, certain patient populations were excluded, as there is insufficient evidence for calculating GRSs in people of certain descents. Examples include: mixed races, West Asians (e.g. Middle Eastern), Native Americans, and Hispanics. Participants with a known *BRCA1* or *BRCA2* mutation were also excluded, as their level of risk for developing breast or prostate cancers is likely higher than the risk conferred by a high GRS.

The study protocol was approved by the NorthShore University HealthSystem Institutional Review Board in Evanston, IL. All participants provided written informed consent.

### Intervention

An aliquot of each participant’s DNA was extracted from the Genomic Health Initiative biorepository and sent to Counsyl, Inc. in San Francisco, CA. Counsyl is a private genomics company that was contracted by NorthShore to sequence patients’ de-identified DNA samples for 299 cancer risk-associated SNPs (Additional file 2: Table 2). A custom panel was developed with templates for the SNP regions.

After DNA samples were collected and analyzed, two GRSs were generated for each participant: a breast cancer GRS and colorectal cancer GRS for each female, and a prostate cancer GRS and colorectal cancer GRS for each male. GRSs were calculated using the equations $\mathrm{GRS}={\prod }_{\mathrm{i}=1}^{\mathrm{n}}\frac{{\mathrm{OR}}_{\mathrm{i}}^{{\mathrm{g}}_{\mathrm{i}}}}{{\mathrm{W}}_{\mathrm{i}}}$ and ${\mathrm{W}}_{\mathrm{i}}= {\mathrm{f}}_{\mathrm{i}}^{2}{\mathrm{OR}}_{\mathrm{i}}^{2}+2{\mathrm{f}}_{\mathrm{i}}(1-{\mathrm{f}}_{\mathrm{i})}{\mathrm{OR}}_{\mathrm{i}}+{(1-{\mathrm{f}}_{\mathrm{i}})}^{2}$, where g_i_ represents the individual’s SNP i genotype (0, 1, or 2 risk alleles), OR_i_ represents the allelic OR of SNP i, and f_i_ represents the risk allele frequency of SNP i in the population.

Data was collected from two sources: questionnaires and participants’ electronic medical records. Participants were asked to respond to three questionnaires timed as follows: before taking the GRS test (i.e. shortly after recruitment), immediately after receiving test results, and three months after results were returned. Questionnaires assessed participants’ feelings about their health, specifically as it related to cancer risk, as well as their plans for breast, prostate and/or colorectal cancer screening. Questionnaires were sent to participants in the format of their choosing: via email using REDCap software, or via postal mail. The questionnaires used were similar to those previously used to collect data on worry about cancer risk and diagnosis, including the US Health Information National Trends Survey (US HINTS), Lerman’s Cancer Worry Scale and the Cancer Worry Scale for Genetic Counseling [12–18]. Cancer screening-related health information was also collected throughout the follow up period of 18–42 months (data not reported).

### Assessment of genetic risk

A GRS is an odds ratio value that confers one’s risk of developing a specific disease relative to average population risk. For example, a woman with a breast cancer GRS equal to 2.0 indicates that she is twice as likely as the average woman to develop breast cancer in her lifetime (~ 26% likelihood vs 12.9%) [19].

GRS values were reported to participants, as well as their category of risk. GRSs in this study were categorized into three groups: low risk, average risk or high risk. Values were categorized as follows, based on varying GRSs corresponding with relative risk and absolute lifetime risks from meta analyses and/or cohort studies: [20–26]

**Table:** 

Cancer type	Low risk GRS	Average risk GRS	High risk GRS
*Breast*	< 0.5 to < 1.0	1.0–1.4	1.5 to > 3.0
*Prostate*	< 0.5 to < 1.0	1.0–1.6	1.7 to > 3.0
*Colorectal*	< 0.5 to < 1.0	1.0–1.6	1.7 to > 3.0

Of note, GRS values for any disease of < 0.5 or > 3.0 were reported as “ < 0.5” or “ > 3.0,” respectively (i.e. exact values for exceptionally low or high scores were not reported).

### Result reporting

GRSs were shared with participants through their electronic medical record portal by their PCPs. PCPs signed a separate consent form to also participate in the study and watched educational videos about GRSs before returning any results. Suggested personalized genomic-based screening guidelines that incorporated GRSs were provided to PCPs for their consideration (Additional file 1: Appendix A).

### Statistical analysis

A t-test or Chi-square test was applied for comparison of demographic and key clinical variables among different groups. P-values for continuous variables (e.g. age) are a p of linear regression, and the p-values for categorical variables (e.g. gender) are a p for the proportion trend test. All statistical analyses were performed using R v3.5.2 [27]. A two-tailed *P* < 0.05 was considered statistically significant.

## Results

### Patient characteristics

The median age of the 281 participants was 58 years. There were more female participants (66.6%). The majority of participants were Caucasian (95.0%), with fewer African American (3.2%) and East Asian (1.8%) participants. No participants had a personal history of breast, prostate or colorectal cancer. Participants reported their FH of breast, prostate and colorectal cancers in first- and second-degree family members. Among all participants (both male and female), 35.2% reported a FH of breast cancer, 25.2% reported a FH of prostate cancer, and 25.6% reported a FH of colorectal cancer (Table 1).

**Table 1:** Demographics

Sex	
Male	94 (33.45%)
Female	187 (66.55%)
Age	
40–49	56 (19.93%)
50–59	94 (33.45%)
60–70	131 (46.62%)
Race	
Caucasian	267 (95.02%)
African American	9 (3.2%)
East Asian	5 (1.78%)
Family history	
Breast cancer	*Missing: 13*
Male	30 (34.09%)
Female	69 (38.33%)
Prostate cancer	*Missing: 12*
Male	24 (26.67%)
Female	47 (26.26%)
Colorectal cancer	*Missing: 8*
Male	23 (25.56%)
Female	49 (26.78%)

### Genetic risk scores and family history

**Fig. 1:**
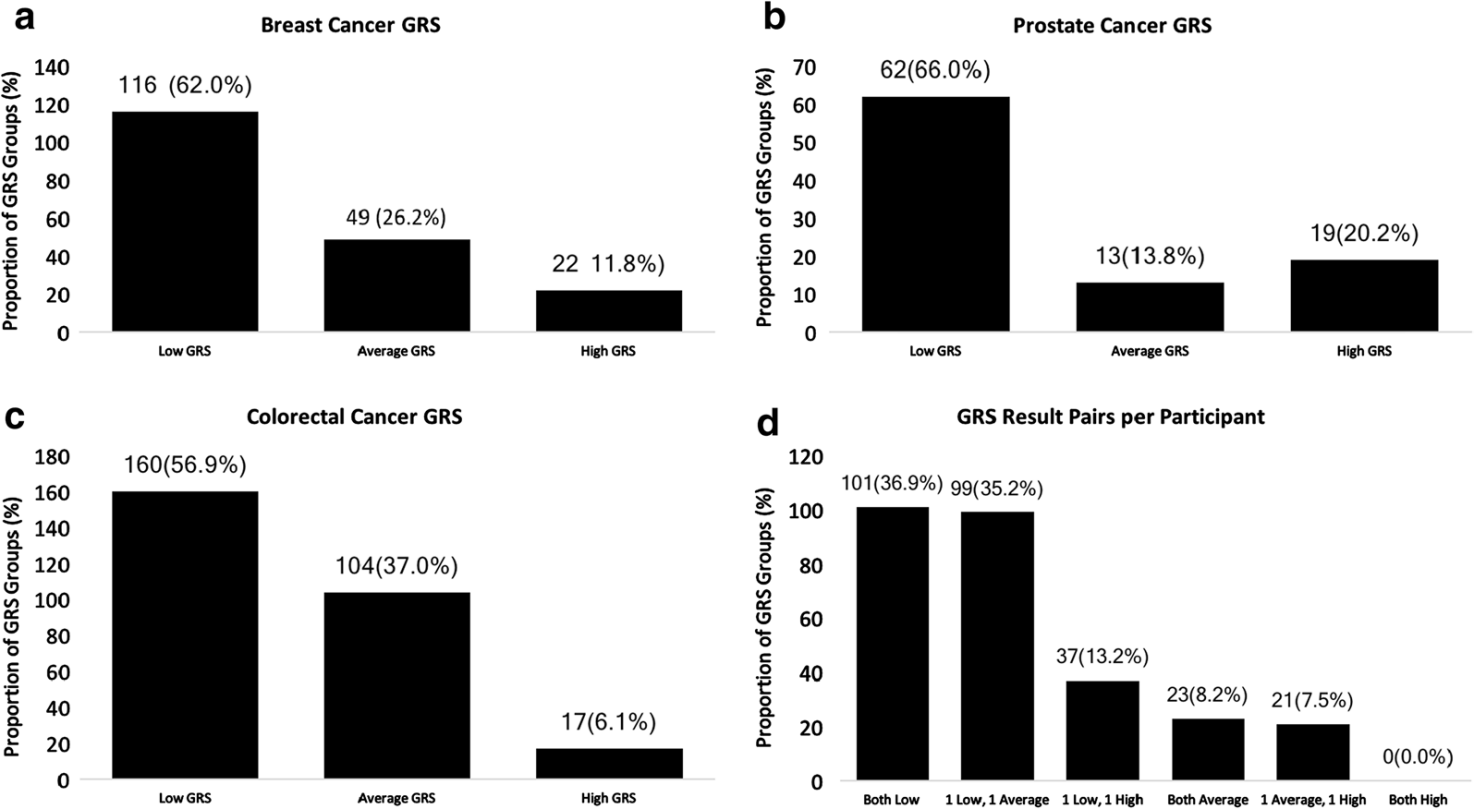
Genetic risk score results

As each participant received 2 GRSs, they can be grouped by their risk category: 101 (36.9%) participants received 2 “low risk” scores, 99 (35.2%) received 1 “low risk” score and 1 “average risk” score, 37 (13.2%) received 1 “low risk” score and 1 “high risk” score, 23 (8.2%) received 2 “average risk” scores, 21 (7.5%) received 1 “average risk” score and 1 “high risk” score, and no one received 2 “high risk” scores (Fig. 1a). For each type of cancer, the majority of participants were found to be “low risk” based on GRS, with 62.0% of breast cancer, 66.0% of prostate cancer, and 56.9% of colorectal cancer GRSs being low risk (Fig. 1a–c). Fewer GRSs for each cancer type were “high risk,” with 11.8% of breast cancer, 20.2% of prostate cancer and 6.1% of colorectal cancer GRSs being high risk (Fig. 1a–c).

We also analyzed the combined risk of GRS with FH within individuals (Additional file 3: Supplemental Figure 1). Among the 180 women with FH information available in this study, 69 (38.33%) reported a family history of breast cancer and 20 (11.11%) had a high GRS for breast cancer. Seven (7) (3.89%) women had both a family history and high GRS for breast cancer. Among the 90 men with FH information available, 24 (26.67%) reported a family history of prostate cancer and 18 (20%) had a high GRS for prostate cancer. Five (5) (5.56%) men had both a family history and high GRS for prostate cancer. Among the 273 women and men with family history information available for colorectal cancer, 72 (26.37%) reported a family history of colorectal cancer and 17 (6.23%) had a high GRS for colorectal cancer. Five (5) (1.83%) had both a family history and high GRS for colorectal cancer.

### Attitudes regarding GRS-related health

Participants were asked to rate the degree to which they agreed with three statements both before and after receiving their GRS results. Response choices, in order of level of agreement, were: “not at all,” “very little,” “somewhat,” “quite a bit,” or “a great deal.”

The three pre-result statements were: (1) I feel anxious when I think about getting my Genetic Risk Scores; (2) I worry about my risk of having cancer when I think about finding out my Genetic Risk Scores; and (3) I feel optimistic regarding my future health. The three corresponding statements that participants responded to after receiving their GRSs were: (1) I feel anxious knowing my Genetic Risk Scores; (2) I worry about my risk of developing cancer; and (3) I feel optimistic regarding my future health.

**Fig. 2:**
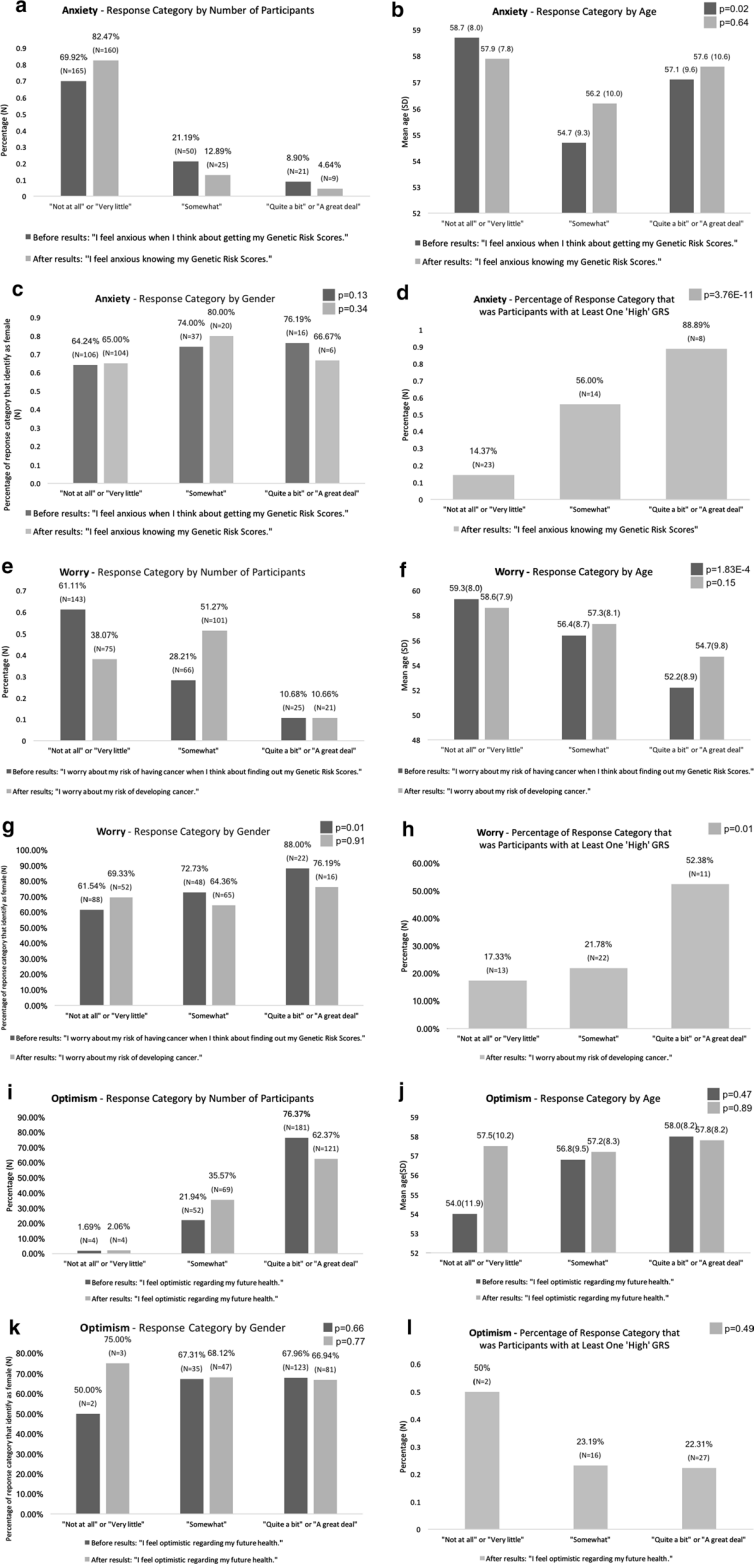
Feelings regarding GRS-Related health before vs after getting GRS results

Before receiving GRSs, younger patients and women reported significantly more worry about risk of developing cancer (Fig. 2). The average age of participants who agreed “quite a bit” or “a great deal” with the statement regarding worrying about cancer was 52.1 years, whereas the average age of those responding “somewhat” was 56.4 years, and those responding “not at all” or “very little” was 59.3 years (p = 1.83E−4). The proportion of women who indicated that they agreed “quite a bit” or “a great deal” to the same statement about worrying about cancer was significantly higher (88.0%) than the proportion of women who stated “somewhat” (72.7%), or “not at all” or “very little” (61.5%) (p = 0.01).

After receiving GRSs, participants who had at least one high GRS reported agreeing significantly more strongly with the statement “I feel anxious knowing my Genetic Risk Scores” than those who had no high GRS (Fig. 2). Of people who responded “quite a bit” or “a great deal,” 88.9% had at least one high GRS, whereas of those who responded “somewhat,” 56% had at least one high GRS, and of those who responded “not at all” or “very little,” only 17.3% had at least one high GRS (p = 3.76E-11). Similarly, those who had at least one reported high GRS agreed significantly more strongly with the statement that they were worried about their risk of developing cancer (p = 0.01) (Fig. 2).

There were no significant differences found between gender, age, or GRS with regards to participants’ reported optimism about their future health neither before nor after receiving GRS results (Fig. 2).

## Self-reported cancer screening plans

**Fig. 3:**
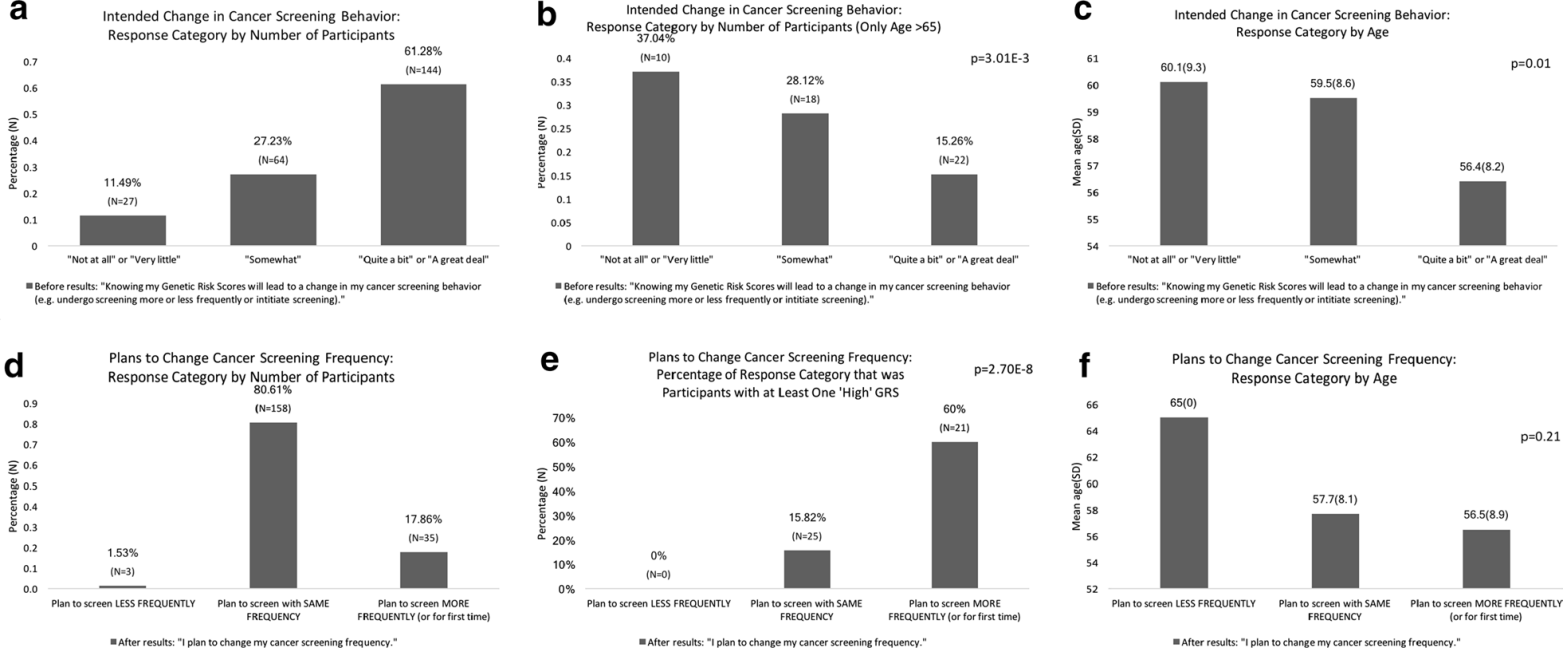
Self-reported plan for cancer screening frequency

Participants were asked broadly to self-report their cancer screening plans. Before receiving GRS results, younger patients were more likely to change their screening behavior based on GRS results (Fig. 3). The average age of those agreeing most strongly was 56.4 years, whereas the average age of those somewhat agreeing was 59.5 years, and those agreeing the least as 60.1 years (p = 0.01).

After receiving GRS results, participants responded to the statement “I plan to change my cancer screening frequency” by saying that they planned to undergo screening less frequently, with the same frequency, or more frequently (or for the first time). Of people who planned to undergo cancer screening more frequently or for the first time, significantly more had at least 1 high GRS (60%) compared with those who planned to undergo cancer screening with the same frequency (15.8%) or less frequently (0%) (p = 2.7E−8) (Fig. 3).

## Discussion

We conducted a clinical utility study of Genetic Risk Scores for cancer risk assessment in a primary care setting. Our goal was to provide a tool that could help further risk stratify patients to ultimately guide more personalized cancer screening plans.

Currently, though colorectal cancer screening recommendations are fairly standard and generally agreed upon by experts, risk is based primarily on family history, recommending earlier and more frequent screening (with colonoscopy) for those with a first- or second-degree family member diagnosed with colorectal cancer [4, 28, 29]. For breast cancer, guidelines regarding screening mammography initiation and frequency have been inconsistent for decades. Currently, the two major guidelines used in primary care are those of the U.S. Preventive Services Task Force (USPSTF) and American Cancer Society. While the American Cancer Society continues to recommend annual mammography screening for all healthy women beginning at age 45, with the option to begin at age 40, the USPSTF now recommends, as of 2009, biannual screening beginning at age 50, unless one has a family history of breast cancer in a first-degree family member [30, 31]. For prostate cancer, a major shift in screening was made in 2012, when the large, U.S., multi-institutional Prostate, Lung, Colorectal, Ovarian (PLCO) clinical trial found prostate specific antigen (PSA) screening to be ineffective in reducing prostate cancer mortality [32, 33]. Based on these results, the USPSTF recommended that PSA screening no longer be performed [34].

Divergent and conflicting recommendations regarding the initiation and frequency of colorectal, breast, and prostate cancer screening leave many individuals unsure about whether, when, and how often to undergo colonoscopy, screening mammography, or PSA testing. Individuals at increased risk are often advised to undergo screening earlier and more often, but these recommendations are largely based on family history, which, if known at all, may be incomplete or inaccurate. Using our GRS risk-prediction model in combination with family history, patients in a primary care setting can better understand their own individual risk for developing colorectal and either breast or prostate cancer. Afforded this new knowledge, we anticipated that patients at increased risk of developing colorectal, breast, and prostate cancer would be motivated to undergo appropriate cancer screening.

We found that most participants (79.4%) were at low or average genetic risk of breast, prostate and colorectal cancers based on their GRS (Fig. 1d). Approximately one-third of participants, however, reported first- or second-degree family histories of these cancers (Table 1). Though both are important to consider in assessing cancer risk and determining screening plans, we found that only 11.3%, 7.5%, and 26.3% of participants who had a positive FH for breast, prostate or colorectal cancers, respectively, also had a high GRS for the respective disease (Additional file 3: Figure 1). Similarly, of participants who had a high GRS for breast, prostate or colorectal cancer, only 53.8, 41.7, and 38.5% of participants reported family history of the disease (Additional file 3: Figure 1). This indicates that GRS may identify a new subset of the population at high risk for these cancers who would not have been identified as high-risk based on current risk-assessment criteria.

Though many participants were deemed low risk of developing one of the three tested cancers based on GRS, a significant concern with regards to the clinical utility of this test was whether participants at high, or even average, risk would suffer anxiety as a result of finding out their GRS. We found that those with a high-risk GRS did report significantly more anxiety, as well as worrying more about developing cancer. Of note, anxiety was measured based on how strongly participants agreed with a statement about having anxiety before and after receiving results rather than a verified anxiety screening questionnaire. To further address this concern, a systematic review was performed by Oliveri et al. to analyze the psychological implications of genetic testing [35]. Regarding genetic testing for cancer risk, they found that levels of anxiety and depression decreased significantly after receiving genetic testing results. However, these studies largely involved *BRCA1* and *BRCA2* testing for breast and ovarian cancers. Though potentially preventable with surgery, considering prophylactic surgery may lead to anxiety. The review also found that knowing results positively affected screening behaviors Alternatively, screening alone can lead to improved health outcomes.

Of note, this study was conducted in a group of particularly motivated patients as evidenced by their participation and follow up in a genetic study. Most had undergone breast (88.2%) or prostate (75.0%) cancer screening within 2 years of this study and/or colorectal cancer (53.5%) within 5 years (data not reported). Thus, it was difficult to assess for increased compliance with recommended cancer screening via EMR data in this population. An important next step in assessing the clinical utility of GRSs should include patient populations with lower cancer screening compliance to assess whether ‘high risk’ scores result in more motivation to undergo cancer screening. Another improvement over the current study design would be a follow-up period greater than 3 years to assess the long-term compliance of participants with information about their genetic risk scores for various cancers.

Another limitation of this study was that, at present, most primary care providers do not regularly incorporate genetics into cancer risk assessment. This is, in part, due to the fact that genetics education targeted at primary care providers is lacking. A review of interventions that provide genetics education for primary care physicians found that receiving short-term genetics education did not necessarily lead to apparent changes in practice [36]. The authors also concluded that there are insufficient studies available to be able to inform, and thus improve, current genetics education tailored to primary care physicians. Further, the lack of established guidelines for PCPs to use in advising patients with known GRSs for the respective cancers is another hurdle for PCPs to incorporate GRSs into their daily practice.

It is encouraging, however, that larger prospective studies are being conducted to further assess the clinical utility of SNP-based risk scores in targeted cancer screening. Specifically, the ongoing WISDOM trial is comparing standard versus risk-based screening to determine onset and frequency of breast cancer screening via mammography for 100,000 women using a polygenic risk score based on 200 SNPs to stratify risk in the risk-based arm [37]. With knowledge provided by studies like this, it is promising that PCPs will be more comfortable utilizing genetics, and more specifically SNP-based risk scores, to guide their practice.

Through the present study, we were able to successfully incorporate genetic risk assessments for specific cancers, in the form of Genetic Risk Scores, into primary care practice. By educating 59 primary care physicians and reporting scores through existing EMR/patient portal workflow, GRSs were successfully reported to 281 patients. As data continues to become available regarding novel cancer-risk associated SNPs, associated with the cancers represented in this study as well as other cancers, we encourage further work to expand the use of Genetic Risk Scores in the primary care setting.

## Conclusions

Genetic risk scores that quantify an individual’s risk of developing breast, prostate and colorectal cancers as compared with a race-defined population average risk have potential clinical utility as a tool for risk stratification and to guide cancer screening in a primary care setting. To maximize their clinical utility and minimize anxiety associated with receiving a high score, we call for improved education programs for primary care providers to increase comfort surrounding the incorporation of genetic testing results into cancer screening plans, thereby empowering patients as a means of helping mitigate possible anxiety.

## Supplementary Information

**Additional file 1.** Appendix A.

**Additional file 3: Table 2.** Risk-associated SNPs used for calculating Genetic Risk Scores.

**Additional file 2: Table 1/Figure 1.** Combined risk of GRS with FH within individuals.

## Data Availability

The datasets generated and/or analyzed during the current study are not publicly available out of protection for individual patient privacy, but de-identified data may be available from the corresponding author on reasonable request.

## Abbreviations

GWASs: Genome-wide association studies
SNPs: Single nucleotide polymorphisms
GRSs: Genetic Risk Scores
FH: Family history
PCPs: Primary care physicians
US HINTS: US Health Information National Trends Survey
USPSTF: The U.S. Preventive Services Task Force
PLCO: Prostate, Lung, Colorectal, Ovarian clinical trial
PSA: Prostate specific antigen
