# Supplementary material for: Assessing the clinical utility of genetic risk scores for targeted cancer screening
Source: J Transl Med. 2021 Jan 22;19:41. doi: 10.1186/s12967-020-02699-w (PMC7821544; doi:10.1186/s12967-020-02699-w)

Appendix A

Recommended screening guidelines incorporating GRS values provided to primary care providers involved in this study.

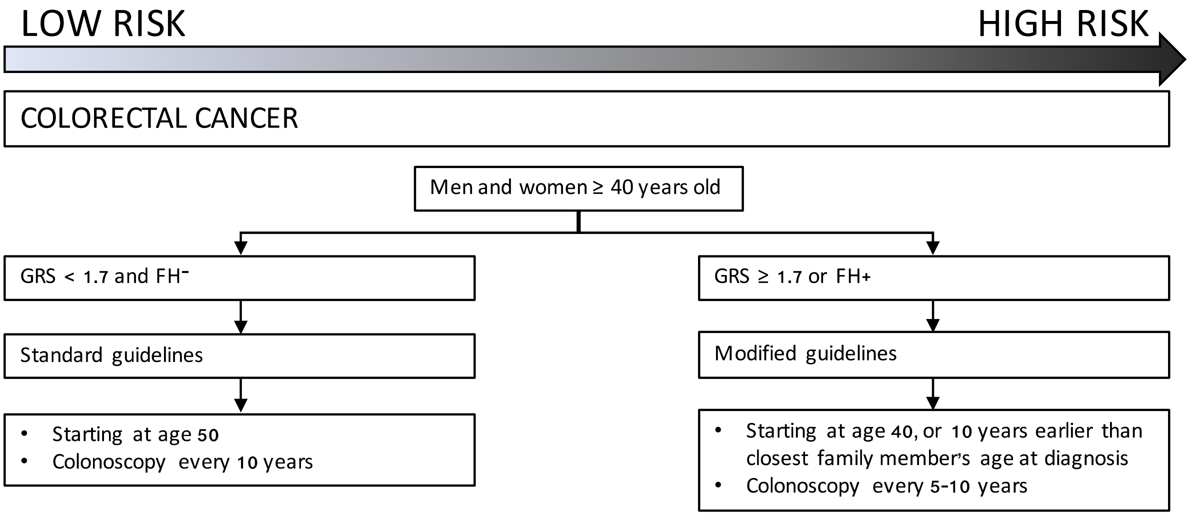

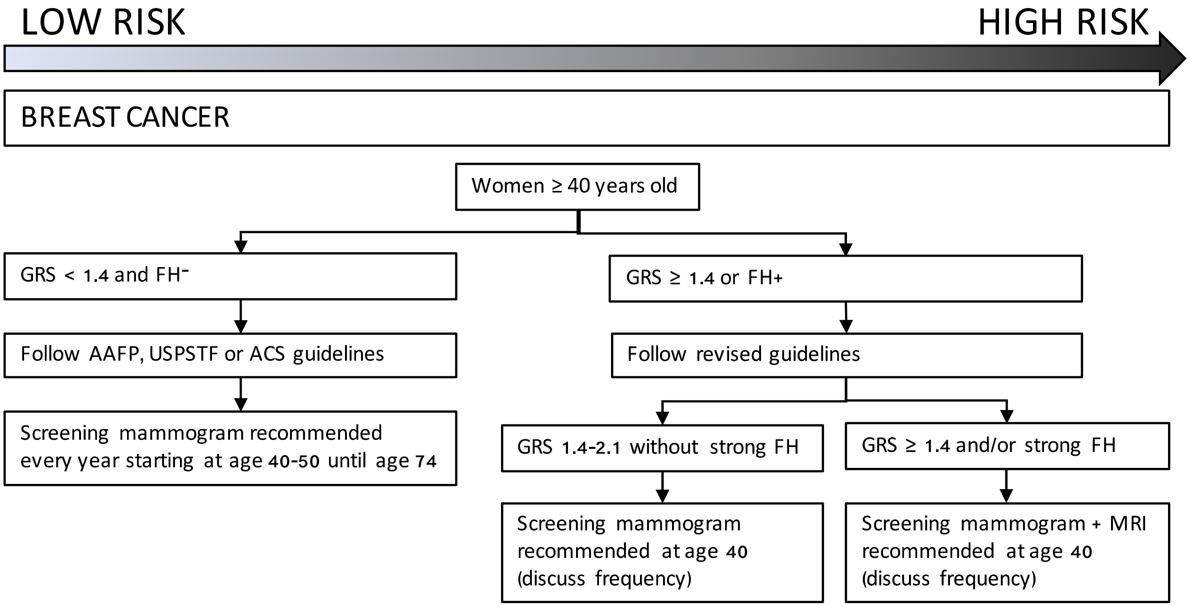

Appendix A (Continued)

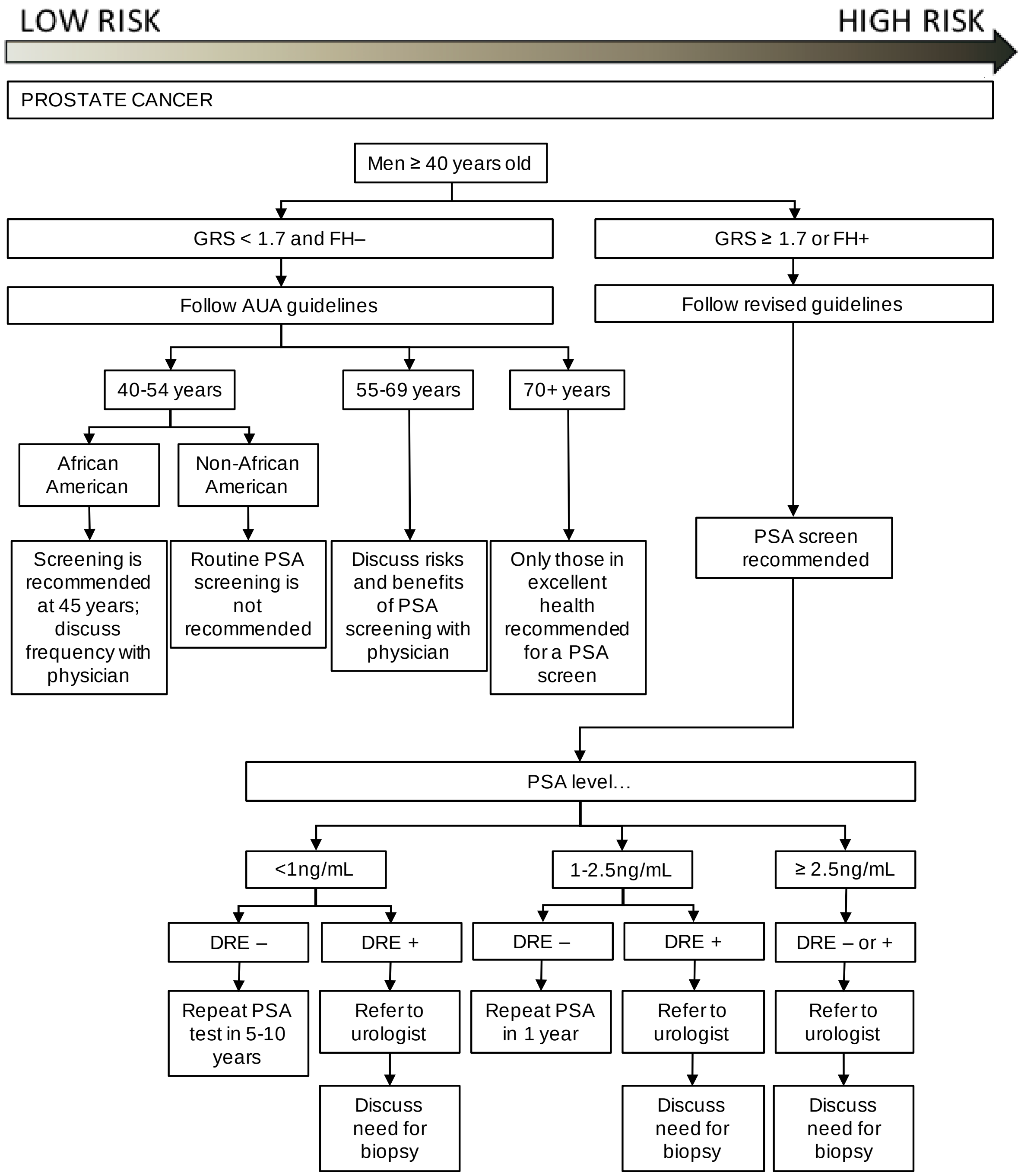

Supplement: Supplementary file 1 — Additional file 1. Appendix A. [file 12967_2020_2699_MOESM1_ESM.pdf]
