# Supplementary material for: Assessing the clinical utility of genetic risk scores for targeted cancer screening
Source: J Transl Med. 2021 Jan 22;19:41. doi: 10.1186/s12967-020-02699-w (PMC7821544; doi:10.1186/s12967-020-02699-w)
Supplement: Supplementary file 2 — Additional file 3: Table 2. Risk-associated SNPs used for calculating Genetic Risk Scores. [file 12967_2020_2699_MOESM3_ESM.pdf]

Supplementary Table 2. Risk-associated SNPs used for calculating Genetic Risk Scores

SNP	CHR	POS	RA	RAF	OR
**Prostate cancer (Caucasian)**					
rs636291	1	10556097	A	0.475272	1.18
rs17599629	1	150658287	G	0.04554	1.1
rs1218582	1	154834183	G	0.183098	1.06
rs4245739	1	204518842	A	0.570025	1.1
rs11902236	2	10117868	T	0.071182	1.07
rs9287719	2	10710730	C	0.203672	1.07
rs1465618	2	43553949	T	0.051076	1.27
rs721048	2	63131731	A	0.029344	1.15
rs10187424	2	85794297	T	0.342927	1.19
rs12621278	2	173311553	A	0.889815	1.35
rs2292884	2	238443226	G	0.053824	1.14
rs3771570	2	242382864	T	0.021141	1.12
rs2660753	3	87110674	T	0.014328	1.18
rs7611694	3	113275624	A	0.320356	1.1
rs10934853	3	128038373	A	0.083868	1.12
rs6763931	3	141102833	A	0.181561	1.18
rs10936632	3	170130102	A	0.267599	1.14
rs10009409	4	73855253	T	0.095914	1.09
rs1894292	4	74349158	G	0.280794	1.1
rs12500426	4	95514609	A	0.211324	1.07
rs7679673	4	106061534	C	0.337561	1.19
rs2121875	5	44365545	C	0.100743	1.09
rs6869841	5	172939426	T	0.043639	1.07
rs4713266	6	11219030	C	0.282067	1.07
rs115457135	6	30073776	A	0.045071	1.08
rs130067	6	31118511	G	0.038848	1.2
rs3096702	6	32192331	A	0.13235	1.07
rs115306967	6	32400939	G	0.458735	1.08
rs1983891	6	41536427	T	0.08151	1.1
rs2273669	6	109285189	G	0.021141	1.07
rs339331	6	117210052	T	0.457517	1.13
rs1933488	6	153441079	A	0.318096	1.12
rs9364554	6	160833664	T	0.07393	1.17
rs12155172	7	20994491	A	0.055319	1.11
rs56232506	7	47437244	A	0.220994	1.07
rs6465657	7	97816327	C	0.227529	1.12
rs2928679	8	23438975	A	0.185502	1.53
rs1512268	8	23526463	T	0.182158	1.23
rs11135910	8	25892142	T	0.021815	1.11

rs12543663	8	127924659	C	0.095172	1.07
rs10086908	8	128011937	T	0.504668	1.25
rs1016343	8	128093297	T	0.042766	1.28
rs13252298	8	128095156	A	0.495475	1.1
rs6983561	8	128106880	C	0.000967	1.61
rs16902094	8	128320346	G	0.024806	1.21
rs445114	8	128323181	T	0.409728	1.1
rs16902104	8	128340908	T	0.024712	1.21
rs7000448	8	128441170	T	0.134689	1.08
rs11986220	8	128531689	A	0.011513	1.56
rs17694493	9	22041998	G	0.018906	1.1
rs76934034	10	46082985	T	0.822649	1.14
rs10993994	10	51549496	T	0.139054	1.57
rs3850699	10	104414221	A	0.461177	1.1
rs4962416	10	126696872	C	0.072092	1.46
rs7127900	11	2233574	A	0.04012	1.28
rs10896449	11	68994667	G	0.235031	1.41
rs11568818	11	102401661	T	0.32707	1.1
rs11214775	11	113807181	G	0.515524	1.08
rs80130819	12	48419618	A	0.824827	1.13
rs10875943	12	49676010	C	0.087675	1.18
rs902774	12	53273904	A	0.021258	1.17
rs1270884	12	114685571	A	0.241769	1.07
rs11839053	13	107063042	C	0.001927	1.54
rs8008270	14	53372330	C	0.653187	1.12
rs7141529	14	69126744	C	0.235031	1.09
rs8014671	14	71092256	G	0.35988	1.07
rs684232	17	618965	C	0.129744	1.1
rs11649743	17	36074979	G	0.66896	1.5
rs7501939	17	36101156	C	0.392502	1.19
rs11650494	17	47345186	A	0.00461	1.15
rs1859962	17	69108753	G	0.237364	1.2
rs7241993	18	76773973	C	0.48986	1.09
rs8102476	19	38735613	C	0.289444	1.12
rs887391	19	41985624	T	0.597374	1.15
rs2735839	19	51364623	G	0.751689	1.2
rs12480328	20	49527922	T	0.869183	1.13
rs2427345	20	61015611	C	0.400689	1.06
rs6062509	20	62362563	T	0.495616	1.12
rs1041449	21	42901421	G	0.196338	1.06
rs2238776	22	19757892	G	0.621732	1.09
rs9623117	22	40452119	C	0.047611	1.18

rs5759167	22	43500212	G	0.253311	1.14
rs2405942	X	9814135	A	0.615126	1.14
rs5945572	X	51229683	A	0.128236	1.07
rs2807031	X	52896949	C	0.0314	1.07
rs5919432	X	67021550	T	0.641921	1.09
rs6625711	X	70139850	A	0.031082	1.07
rs4844289	X	70407983	G	0.144172	1.05
**Prostate cancer (African American)**					
rs4245739	1	204518842	A	0.595058	1.09
rs10187424	2	85794297	T	0.125387	0.833333
rs12621278	2	173311553	A	0.973577	1.48
rs2292884	2	238443226	G	0.316406	1.1
rs7679673	4	106061534	C	0.147994	1.14
rs12653946	5	1895829	T	0.15992	1.09
rs130067	6	31118511	G	0.036941	0.82
rs1983891	6	41536427	T	0.232613	1.12
rs339331	6	117210052	T	0.583543	1.19
rs9364554	6	160833664	T	0.004369	1.18
rs10486567	7	27976563	G	0.511654	1.19
rs56232506	7	47437244	A	0.01199	1.29
rs1512268	8	23526463	T	0.416799	0.862069
rs12543663	8	127924659	A	0.743561	1.162791
rs10086908	8	128011937	T	0.575171	1.28
rs13252298	8	128095156	A	0.868065	1.15
rs6983561	8	128106880	C	0.198025	1.29
rs116041037	8	128131809	A	0.000949	2.67
rs445114	8	128323181	T	0.1133	1.09
rs6983267	8	128413305	G	0.80425	1.33
rs7000448	8	128441170	T	0.450509	1.14
rs11986220	8	128531689	A	0.003931	1.38
rs817826	9	110156300	C	0.088506	1.08
rs10993994	10	51549496	T	0.369542	1.1
rs7127900	11	2233574	A	0.134396	1.1
rs10896449	11	68994667	G	0.478034	1.15
rs10875943	12	49676010	C	0.397404	1.15
rs1270884	12	114685571	A	0.036902	1.22
rs684232	17	618965	C	0.43178	0.925926
rs11650494	17	47345186	A	0.055413	1.1
rs7210100	17	47436749	A	0.003158	1.51
rs8102476	19	38735613	C	0.565504	1.08
rs5759167	22	43500212	G	0.568667	1.15
rs5945572	X	51229683	A	0.081796	1.08

rs5919432	X	67021550	T	0.146153	0.900901
**Prostate cancer (East Asian)**					
rs13385191	2	20888265	G	0.162812	1.1
rs1465618	2	43553949	T	0.541696	1.17
rs721048	2	63131731	A	0.001624	1.39
rs10187424	2	85794297	T	0.362645	1.12
rs12621278	2	173311553	A	0.523452	1.14
rs2660753	3	87110674	T	0.054429	1.19
rs2055109	3	87467332	C	0.00257	1.29
rs7611694	3	113275624	A	0.096162	1.16
rs6763931	3	141102833	A	0.102208	1.11
rs7679673	4	106061534	C	0.041209	1.16
rs12653946	5	1895829	T	0.104717	1.26
rs1983891	6	41536427	T	0.104329	1.15
rs339331	6	117210052	T	0.411651	1.23
rs9364554	6	160833664	C	0.494209	1.1
rs12155172	7	20994491	A	0.065331	1.14
rs10486567	7	27976563	G	0.014352	1.15
rs6465657	7	97816327	C	0.742527	1.14
rs1512268	8	23526463	T	0.087143	1.32
rs10086908	8	128011937	T	0.67947	1.2
rs13252298	8	128095156	A	0.437318	1.38
rs16901979	8	128124916	A	0.06938	1.39
rs620861	8	128335673	G	0.32707	1.13
rs6983267	8	128413305	G	0.167526	1.23
rs1447295	8	128485038	A	0.020678	1.54
rs817826	9	110156300	C	0.012078	1.49
rs10993994	10	51549496	T	0.236002	1.2
rs3850699	10	104414221	A	0.633934	1.17
rs2252004	10	122844709	C	0.48567	1.17
rs12791447	11	7556577	G	0.012522	1.23
rs1938781	11	58915110	G	0.093452	1.17
rs11568818	11	102401661	T	0.852668	1.21
rs10875943	12	49676010	C	0.732051	1.15
rs9600079	13	73728139	T	0.222878	1.24
rs7153648	14	61122526	C	0.029515	1.15
rs58262369	14	64693912	T	0.010878	1.28
rs11649743	17	36074979	G	0.445289	1.18
rs4430796	17	36098040	A	0.514519	1.23
rs7241993	18	76773973	C	0.32982	1.14
rs887391	19	41985624	T	0.36857	1.1
rs103294	19	54797848	C	0.257455	1.34

rs5759167	22	43500212	G	0.473482	1.15
**Breast cancer (Caucasian)**					
rs616488	1	10566215	A	0.455355	1.06
rs11552449	1	114448389	T	0.030276	1.07
rs11249433	1	121280613	G	0.151165	1.09
rs12405132	1	145644984	C	0.378102	1.05
rs12048493	1	149927034	C	0.147686	1.07
rs72755295	1	242034263	G	0.001005	1.15
rs12710696	2	19320803	T	0.128881	1.1
rs4849887	2	121245122	C	0.820836	1.1
rs2016394	2	172972971	G	0.272797	1.05
rs1550623	2	174212894	A	0.721141	1.06
rs1045485	2	202149589	G	0.763701	1.03
rs13387042	2	217905832	A	0.271754	1.14
rs16857609	2	218296508	T	0.063454	1.08
rs6762644	3	4742276	G	0.1444	1.07
rs4973768	3	27416013	T	0.221558	1.11
rs12493607	3	30682939	C	0.130755	1.06
rs6796502	3	46866866	G	0.807662	1.09
rs1053338	3	63967900	G	0.01486	1.08
rs9790517	4	106084778	T	0.057648	1.05
rs6828523	4	175846426	C	0.770357	1.11
rs10069690	5	1279790	T	0.070437	1.06
rs2736108	5	1297488	C	0.518976	1.06
rs13162653	5	16187528	G	0.286332	1.05
rs2012709	5	32567732	T	0.236682	1.05
rs10941679	5	44706498	G	0.0661	1.12
rs889312	5	56031884	C	0.08191	1.12
rs2229882	5	56168712	T	0.002247	1.45
rs10472076	5	58184061	C	0.145466	1.05
rs1353747	5	58337481	T	0.80946	1.09
rs7707921	5	81538046	A	0.56716	1.08
rs1432679	5	158244083	C	0.180285	1.07
rs11242675	6	1318878	T	0.387382	1.04
rs204247	6	13722523	G	0.191581	1.05
rs9257408	6	28926220	C	0.143111	1.05
rs17529111	6	82128386	C	0.046354	1.06
rs2180341	6	127600630	G	0.064567	1.41
rs12662670	6	151918856	G	0.004556	1.17
rs2046210	6	151948366	A	0.104071	1.08
rs6964587	7	91630620	T	0.143035	1.05
rs4593472	7	130667121	C	0.425887	1.05

rs720475	7	144074929	G	0.569572	1.06
rs9693444	8	29509616	A	0.100552	1.07
rs13365225	8	36858483	A	0.651895	1.05
rs6472903	8	76230301	T	0.684094	1.1
rs2943559	8	76417937	G	0.004928	1.13
rs13267382	8	117209548	A	0.137641	1.05
rs13281615	8	128355618	G	0.177157	1.09
rs11780156	8	129194641	T	0.029618	1.07
rs1011970	9	22062134	T	0.028359	1.06
rs10759243	9	110306115	A	0.085322	1.05
rs865686	9	110888478	T	0.412549	1.2
rs2380205	10	5886734	C	0.303821	1.06
rs7072776	10	22032942	A	0.079863	1.07
rs11814448	10	22315843	C	0.000471	1.27
rs10995190	10	64278682	G	0.69289	1.16
rs704010	10	80841148	T	0.136013	1.08
rs7904519	10	114773927	G	0.219492	1.05
rs11199914	10	123093901	C	0.465397	1.05
rs2981579	10	123337335	A	0.158643	1.27
rs3817198	11	1909006	C	0.092903	1.07
rs3903072	11	65583066	G	0.263066	1.05
rs614367	11	69328764	T	0.025889	1.21
rs78540526	11	69331418	T	0.007621	1.34
rs554219	11	69331642	G	0.017583	1.26
rs11820646	11	129461171	C	0.385144	1.05
rs12422552	12	14413931	C	0.058903	1.04
rs10771399	12	28155080	A	0.790321	1.16
rs17356907	12	96027759	A	0.486646	1.1
rs1292011	12	115836522	A	0.337096	1.09
rs2236007	14	37132769	G	0.610899	1.08
rs2588809	14	68660428	T	0.021638	1.08
rs999737	14	69034682	C	0.578512	1.09
rs941764	14	91841069	G	0.119163	1.07
rs11627032	14	93104072	T	0.558457	1.06
rs8051542	16	52534167	T	0.2053	1.09
rs3803662	16	52586341	A	0.078008	1.24
rs3112612	16	52635164	A	0.176988	1.15
rs17817449	16	53813367	T	0.3312	1.08
rs13329835	16	80650805	G	0.051438	1.08
rs6504950	17	53056471	G	0.528674	1.06
rs745570	17	77781725	A	0.243838	1.05
rs527616	18	24337424	G	0.370272	1.05

rs1436904	18	24570667	T	0.372832	1.04
rs6507583	18	42399590	A	0.876658	1.1
rs3745185	19	17384267	G	0.296698	1.16
rs2363956	19	17394124	T	0.22553	1.03
rs4808801	19	18571141	A	0.428763	1.08
rs3760982	19	44286513	A	0.212982	1.05
rs2284378	20	32588095	C	0.461448	1.05
rs2823093	21	16520832	G	0.536556	1.09
rs132390	22	29621477	C	0.00105	1.14
rs6001930	22	40876234	C	0.014066	1.12
**Breast cancer (African American)**					
rs4849887	2	121245122	C	0.473069	1.16
rs13387042	2	217905832	A	0.540372	1.12
rs16857609	2	218296508	T	0.056596	1.17
rs10069690	5	1279790	T	0.345979	1.13
rs204247	6	13722523	G	0.121592	1.13
rs2981579	10	123337335	A	0.371246	1.18
rs2981578	10	123340311	C	0.751516	1.28
rs999737	14	69034682	C	0.916423	1.59
rs8051542	16	52534167	T	0.098094	1.4
rs17817449	16	53813367	T	0.362284	1.21
rs2363956	19	17394124	T	0.25	1.14
**Breast cancer (East Asian)**					
rs13393577	2	213296863	C	0.00252	1.53
rs16857609	2	218296508	T	0.370394	1.12
rs4973768	3	27416013	T	0.033893	1.12
rs6788895	3	150467808	G	0.396018	1.22
rs4415084	5	44662515	C	0.190009	1.12
rs889312	5	56031884	C	0.329361	1.16
rs1419026	6	82089179	T	0.079919	1.07
rs9485372	6	149608874	G	0.343982	1.11
rs2046210	6	151948366	A	0.13061	1.28
rs9693444	8	29509616	A	0.087261	1.15
rs10822013	10	64251977	T	0.235322	1.12
rs941827	10	114558887	T	0.601866	1.05
rs2981578	10	123340311	C	0.304704	1.23
rs17221259	12	14410485	C	0.057648	1.25
rs3803662	16	52586341	A	0.412036	1.21
rs4784227	16	52599188	T	0.061901	1.25
**Colorectal cancer (Caucasian)**					
rs10911251	1	183081194	A	0.328329	1.09
rs6691170	1	222045446	T	0.137196	1.06

rs6687758	1	222164948	G	0.043222	1.09
rs11903757	2	192587204	C	0.025664	1.16
rs992157	2	219154781	A	0.322056	1.1
rs10936599	3	169492101	C	0.563851	1.04
rs367615	5	108948937	T	0.651733	1.35
rs647161	5	134499092	A	0.435732	1.07
rs1321311	6	36622900	A	0.055838	1.1
rs16892766	8	117630683	C	0.006416	1.27
rs7014346	8	128424792	A	0.131696	1.19
rs10795668	10	8701219	A	0.10106	1.12
rs704017	10	80819132	G	0.333968	1.06
rs1035209	10	101345366	T	0.038025	1.13
rs1535	11	61597972	A	0.431518	1.07
rs3824999	11	74345550	G	0.246214	1.08
rs3802842	11	111171709	C	0.076895	1.11
rs3217810	12	4388271	T	0.016667	1.19
rs3217901	12	4405389	G	0.180285	1.1
rs7136702	12	50880216	T	0.113502	1.06
rs11169552	12	51155663	C	0.500132	1.09
rs59336	12	115116352	T	0.2494	1.09
rs4444235	14	54410919	C	0.200883	1.11
rs1957636	14	54560018	T	0.15437	1.08
rs4779584	15	32994756	C	0.637123	1.18
rs9929218	16	68820946	G	0.510082	1.1
rs4939827	18	46453463	T	0.267186	1.2
rs10411210	19	33532300	C	0.803892	1.15
rs961253	20	6404281	A	0.121383	1.12
rs4813802	20	6699595	G	0.12468	1.09
rs2423279	20	7812350	C	0.061405	1.07
rs4925386	20	60921044	C	0.513946	1.08
rs5934683	X	9751474	T	0.11813	1.07
**Colorectal cancer (African American)**					
rs647161	5	134499092	A	0.315844	1.14
rs16892766	8	117630683	C	0.016358	1.18
rs6983267	8	128413305	G	0.80425	1.2
rs3824999	11	74345550	G	0.027956	1.15
**Colorectal cancer (East Asian)**					
rs6687758	1	222164948	G	0.042312	1.12
rs647161	5	134499092	A	0.115532	1.15
rs1321311	6	36622900	A	0.03393	1.09
rs7758229	6	160840252	T	0.054569	1.28
rs6983267	8	128413305	G	0.167526	1.14

rs10795668	10	8701219	G	0.405387	1.15
rs704017	10	80819132	G	0.080713	1.1
rs11196172	10	114726843	A	0.448364	1.14
rs1535	11	61597972	A	0.18122	1.15
rs3824999	11	74345550	G	0.156816	1.06
rs10774214	12	4368352	T	0.0961	1.17
rs10849432	12	6385727	T	0.619999	1.14
rs2238126	12	12009741	G	0.26061	1.17
rs4779584	15	32994756	T	0.637283	1.12
rs12603526	17	800593	C	0.060025	1.1
rs7229639	18	46450976	A	0.022741	1.2
rs4939827	18	46453463	T	0.095666	1.12
rs10411210	19	33532300	C	0.64497	1.15
rs1800469	19	41860296	G	0.177662	1.09
rs2423279	20	7812350	C	0.106341	1.13

Abbreviations: SNP, single nucleotide polymorphisms; CHR, chromosome; POS, position; RA, risk allele; RAF, risk allele frequency; OR, odds ratio.
