# Supplementary figures and images for: Assessing the clinical utility of genetic risk scores for targeted cancer screening
Source: J Transl Med. 2021 Jan 22;19:41. doi: 10.1186/s12967-020-02699-w (PMC7821544; doi:10.1186/s12967-020-02699-w)

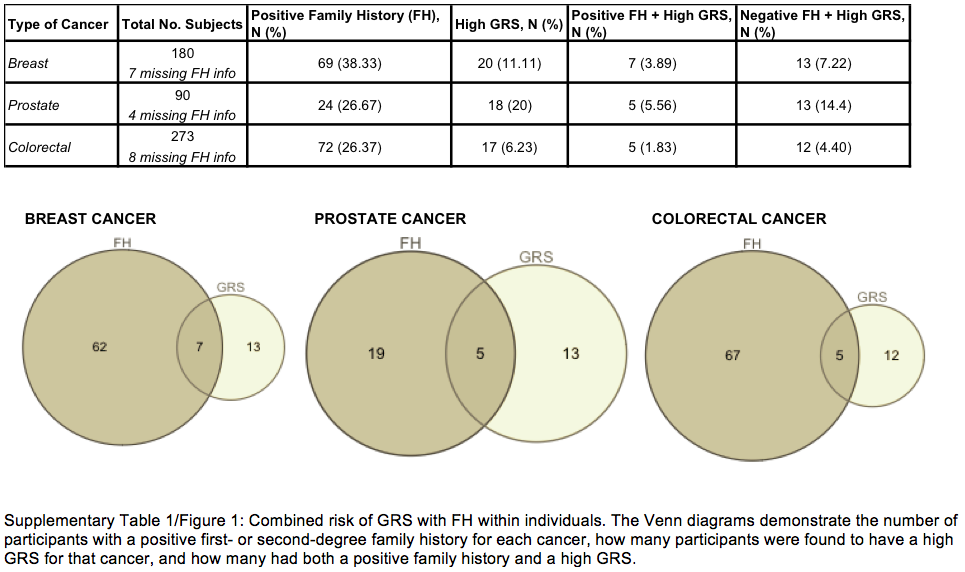

Supplement: Supplementary file 3 — Additional file 2: Table 1/Figure 1. Combined risk of GRS with FH within individuals. [file 12967_2020_2699_MOESM2_ESM.png]
